# Analysis of α-syn and *parkin* interaction in mediating neuronal death in *Drosophila* model of Parkinson's disease

**DOI:** 10.3389/fncel.2023.1295805

**Published:** 2024-01-04

**Authors:** Sonia Narwal, Amit Singh, Meghana Tare

**Affiliations:** ^1^Department of Biological Sciences, Birla Institute of Technology and Science, Pilani, Rajasthan, India; ^2^Department of Biology, University of Dayton, Dayton, OH, United States

**Keywords:** α-synuclein, *parkin*, mitochondrial morphology, dopaminergic neurons, Parkinson's disease, tyrosine hydroxylase, *Drosophila melanogaster*

## Abstract

One of the hallmarks of Parkinson's Disease (PD) is aggregation of incorrectly folded α-synuclein (*SNCA*) protein resulting in selective death of dopaminergic neurons. Another form of PD is characterized by the loss-of-function of an E3-ubiquitin ligase, *parkin*. Mutations in *SNCA* and *parkin* result in impaired mitochondrial morphology, causing loss of dopaminergic neurons. Despite extensive research on the individual effects of *SNCA* and *parkin*, their interactions in dopaminergic neurons remain understudied. Here we employ *Drosophila* model to study the effect of collective overexpression of *SNCA* along with the downregulation of *parkin* in the dopaminergic neurons of the posterior brain. We found that overexpression of *SNCA* along with downregulation of *parkin* causes a reduction in the number of dopaminergic neuronal clusters in the posterior region of the adult brain, which is manifested as progressive locomotor dysfunction. Overexpression of *SNCA* and downregulation of *parkin* collectively results in altered mitochondrial morphology in a cluster-specific manner, only in a subset of dopaminergic neurons of the brain. Further, we found that *SNCA* overexpression causes transcriptional downregulation of *parkin*. However, this downregulation is not further enhanced upon collective *SNCA* overexpression and *parkin* downregulation. This suggests that the interactions of *SNCA* and *parkin* may not be additive. Our study thus provides insights into a potential link between α*-synuclein* and *parkin* interactions. These interactions result in altered mitochondrial morphology in a cluster-specific manner for dopaminergic neurons over a time, thus unraveling the molecular interactions involved in the etiology of Parkinson's Disease.

## Introduction

Parkinson's Disease (PD), first characterized by James Parkinson in 1817 (Jost and Reichmann, 2017), is the second most common persistent movement neurodegenerative disorder, after Alzheimer's disease, with no cure to date. PD affects 0.8% of the worldwide population among other neurological disorders (Ray Dorsey et al., 2018). It is characterized by the specific loss of dopaminergic (DA) neurons in the substantia nigra pars compacta (SNpc) region of the midbrain which leads to motor symptoms including bradykinesia, muscular rigidity, resting tremor, postural instability (Wood-Kaczmar et al., 2006; Chartier and Duyckaerts, 2018). Progressive loss of DA neurons and the presence of intra-neuronal Lewy bodies (mainly aggregation of α-synuclein protein) are majorly considered neuropathological features of PD (Chartier and Duyckaerts, 2018; Shahmoradian et al., 2019).

*SNCA* and *parkin* are the two major genes involved in both sporadic as well as genetic forms of PD (Corti et al., 2011). *SNCA* encodes an α-synuclein protein and mutations in *SNCA* are associated with the Autosomal-Dominant form of PD (Polymeropoulos et al., 1997; Kachergus et al., 2004; Appel-Cresswell et al., 2013). Different *in-vitro* and *in-vivo* studies have shown that misfolded α-synuclein aggregation causes neurotoxicity by influencing the neurotransmission, synaptic vesicle exocytosis, recycling as well as endocytosis in the substantia nigra region. Migration of α-synuclein between neurons in a prion-like manner to propagate the formation of Lewy bodies throughout the substantia nigra has also been suggested (Li et al., 2008; Burré et al., 2010; Olanow and Brundin, 2013). *Parkin* encodes an E3 ubiquitin ligase and is the second most common cause of autosomal recessive early-onset PD (Kitada et al., 1998). *Parkin* loss-of-function causes neurodegeneration with or without forming Lewy bodies in PD patients (Yokochi, 1997; Pramstaller et al., 2005; Johansen et al., 2018). Studies have reported that overexpression of *parkin* results in reduced neurotoxicity caused by α-synuclein in different models (Oluwatosin-Chigbu et al., 2003; Haywood and Staveley, 2004; Khandelwal et al., 2010). Studies have also reported that *parkin* mutation results in no aggregation of α-synuclein in mice (Goldberg et al., 2003; Van Rompuy et al., 2015). However, very limited *in-vivo* studies have been done to explore the link between *SNCA* and *parkin*.

At the cellular level, mitochondria dysfunction has been considered a major hallmark of PD (Nicoletti et al., 2021). Studies in different model systems have shown that α-synuclein causes mitochondria fragmentation, disturbed membrane potential, complex I deficits, and reduced ATP production. In *Drosophila*, elongated as well as fragmented mitochondria have been reported due to α*-synuclein* overexpression (Nakamura, 2013; Ordonez et al., 2018; Krzystek et al., 2021). Loss-of-function mutation in *parkin* has also shown the mitochondrial pathology demonstrated as mitochondrial elongation, swelling, and cristae disruption in *in-vitro* and *in-vivo* models (Greene et al., 2003; Pesah et al., 2004; Deng et al., 2008; Yu et al., 2011; Noda et al., 2020). However, fused and fragmented mitochondria have also been reported in DA neurons of *parkin* mutant *Drosophila* (Cackovic et al., 2018). Moreover, it has been found that mitochondrial fragmentation caused by α-synuclein overexpression can be rescued by co-expression of *parkin*, PINK1, or DJ-1, indicating that α-synuclein and parkin may function in the same pathway (Kamp et al., 2010; reviewed in Jeśko et al., 2019). However, limited *in-vivo* studies have been done to test the effect of the interaction of *SNCA* and *parki*n on mitochondrial morphology in Parkinson's disease.

In this report, we have tested the interaction of *parkin* and *SNCA* and their effect on mitochondria in DA neurons using a humanized *Drosophila melanogaster* model of PD. *Drosophila* provides a simple, yet powerful *in-vivo* system to study neurodegenerative diseases including Parkinson's disease. It has a compact genome size (1/30th of the human genome), limited genetic redundancy, and a shorter generation time (12–15 days) and life span (60–80 days) (Bier, 2005). Almost 75% of all human disease genes are well conserved in the *Drosophila* genome sequence (Dawson et al., 2010; Aryal and Lee, 2019). Although *Drosophila* does not have an *SNCA* homolog, it mimics major PD symptoms i.e., locomotor dysfunction, age-dependent DA neuronal loss, and Lewy bodies aggregation upon overexpression of human *SNCA* using the GAL4/UAS system (Feany and Bender, 2000). *Drosophila* adult fly brain contains approximately 100 DA neurons, which are grouped into different clusters according to their anatomical position: PAL (paired anterior lateral), PAM (paired anterior medial), PPM1/2 and PPM3 (paired posterior medial), and PPL1 and PPL2 (paired posterior lateral) (Monastirioti, 1999; Mao and Davis, 2009). It has been suggested that each cluster-specific DA neuron projects to distinct functional areas of the brain (White et al., 2010), although the function of each of the DA clusters is not completely explored. PPL1 and PPM3 are more explored clusters in the case of PD using animal models because these are functionally homologous to the mammalian substantia nigra pars compacta region (Strausfeld and Hirth, 2013). It has been shown that PPL1 is associated largely with memory formation (Heisenberg, 2003) whereas, PPM3 is reported to be a center for the control of locomotor behavior (Strauss, 2002). In PD models of *Drosophila*, different clusters have been reported to be affected in a context-dependent manner.

In this study, we have used GAL4/UAS system specific for DA neurons to understand DA neuronal loss because of accumulation of α*-* s*ynuclein* as well as downregulation of *parkin* in a time-dependent manner in adult fly brain, mimicking the PD in humans. We found DA neuronal loss upon genetic alterations is cluster-specific, exhibiting altered mitochondrial morphology. Our studies have implications in providing leads for cellular and molecular interactions on DA neuronal degeneration during the onset and progression of PD.

## Methods

### Fly strains

All fly stocks, genetic crosses, and F1 progenies were maintained on standard fly food containing agar, maize powder, yeast, and sugar at 25°C. The GAL4/UAS system was used to obtain the desired genotype for the experiments (Brand and Perrimon, 1993; Tare et al., 2011). Transgenic *Drosophila* lines: *UAS-GFP* (a kind gift from S.C Lakhotia's Lab)*, UAS-Mito-HA-GFP.AP* (BL-8442), *UAS-Hsap/SNCA.F* (BL-8146), *UAS-SNCA.J}1/CyO* (BL-51375), *UAS-Park*^*RNAi*^ (BL-37509) were used. Driver Gal4 line *TH-Gal4* or *ple-GAL4* (BL-8848) was used to overexpress or downregulate the responder lines of genes in DA neurons.

### Climbing assays

To determine locomotor activity, climbing assays were performed (Pendleton et al., 2002). Ten flies per genotype were transferred into a cylindrical glass tube after anesthetization and left for 5–10 min for revival and acclimatization at room temperature. Tubes were marked up to 8 cm above the bottom of the vial. After acclimatization, flies were gently tapped down to the bottom of the vial and the number of flies that crossed the 8 cm mark was recorded after 10 s. Three trials were performed, and numbers were then averaged, and the resulting mean was used as the overall value for each single group of flies. For all genotypes, three replicates were carried out.

### Quantitative real-time PCR

Total RNA was extracted from 25 to 30 fly heads using the TRIzol method (Invitrogen) specified in the Jove protocol (URL: https://www.jove.com/video/50245) (Mehta et al., 2019). RNA concentrations were measured with a Nanodrop ND-1000 Spectrophotometer and equal amounts of RNA were reverse transcribed using Verso cDNA Synthesis Kit (AB1453A). qPCR was performed using 5x HOT FIREPol^®^ EvaGreen^®^ qPCR Mix Plus (Solis BioDyne). *RP49* was used as the internal control gene. Primer sequences were as follows:

***RP49*** Forward: 5′-CCAAGGACTTCATCCGCCACC-3′

Reverse: 5′- GCGGGTGCGCTTGTTCGATCC-3′

***Parkin*** Forward: 5′-ATTTGCCGGTAAGGAACTAAG C-3′

Reverse: 5′-AAGTGGCCGACTGGATTTTCT-3′

### Adult brain immunohistochemistry

Brain preparation for confocal microscopy imaging was done as described by Tito et al. (2016). Briefly, Adult brains of the desired genotype were dissected in cold 1X PBS and incubated with fixative solution (4% formaldehyde) in 1XPBST (0.1%TritonX-100) for 20 min at room temperature. After three washes with 1XPBST for 10 min each wash, blocking was done using 1% BSA for 1hr at room temperature. Brains were probed with rabbit anti-TH (#AB152) at 1:1,000, overnight (12–16) at 4°C. Following three washes for 10 min, brains were incubated with Goat anti-Rabbit, Alexa-Fluor Plus 555 (**#**A32732) (1:4,000) secondary antibodies at room temperature for 2 h. Brains were washed three times for 15 min, then they were mounted between two glass coverslips by using an antifade medium on microscope slides. A confocal microscope was used to acquire z-stacks at 1 μm intervals with 20 × /N.A.0.60 Plan-Apochromat objective. The number of TH-positive neurons was counted manually within each cluster of posterior regions of the brains.

### Immunoblotting

Western blot was performed as previously described (Gogia et al., 2017) with some modifications. F1 progenies of the desired genotype were collected in 1.5 ml Eppendorf tubes and snap-frozen in liquid nitrogen. *Drosophila* heads (~30–50) were decapitated and homogenized in 100 ul 1x RIPA buffer (Merck, #20–188) containing 1% protease inhibitor (Sigma, P8340) using a sterilized pestle. The homogenates were centrifuged at 10,000 rpm at 4 C for 10 min. The supernatant was collected and assayed for protein concentration. The protein (80 μg) was resolved on 12% SDA-PAGE and then transferred to 0.2 μm nitrocellulose membrane (Bio Rad, #1620112). After blocking the membrane with 3% BSA in TBS-T (0.05%Tween-20), the membrane was incubated overnight at 4 C with primary antibodies. The primary antibodies used were rabbit anti-Drosophila Parkin (Merck, SAB1300355, 1:500), mouse E7 anti-beta tubulin (DSHB-S1-810, 1:200). Following three washes with TBS-T, the membrane was incubated with appropriate HRP-conjugated secondary antibodies: goat anti-mouse (Thermo Scientific # 31430, 1:1,000), mouse anti-Rabbit (GenScript, #A01856, 1:1,000) for 2-h at room temperature and the signal was detected using ECL substrate (Bio Rad #1705061). Image analysis and quantification was done using ImageJ software. Western blot was done on the same membranes after stripping between each application of the antibody.

### Mitochondrial morphology measurement

To assess the mitochondrial morphology in DA neurons, the *UAS-MitoGFP* fly strain was used to tag the mitochondria, and DA neurons were stained using anti TH antibody. Z-stack of one PPL1 and one PPM3 DA neuronal cluster per brain was imaged using confocal microscope at 63 × /N.A.1.30 oil with 1.5 zoom. A total of four brains per genotype were scanned. We used publicly available ImageJ Mito-Morphology Macro created by Dagda et al. (2009), to quantify the mitochondria. Average area and circularity were calculated representing elongation and fragmentation of mitochondria respectively.

### Statistical analysis

GraphPad Prism 8.0.1 was used for statistical analysis and graphical display of the data. Significance is expressed as *p* values which were determined with one-way ANOVA and Two-way ANOVA followed by Tukey's multiple comparison tests as indicated in the figure legends.

Each result is representative of at least three biological and three technical repeats.

## Results

### *SNCA* overexpression and *parkin* downregulation together exhibit locomotor dysfunctions

**Figure 1:**
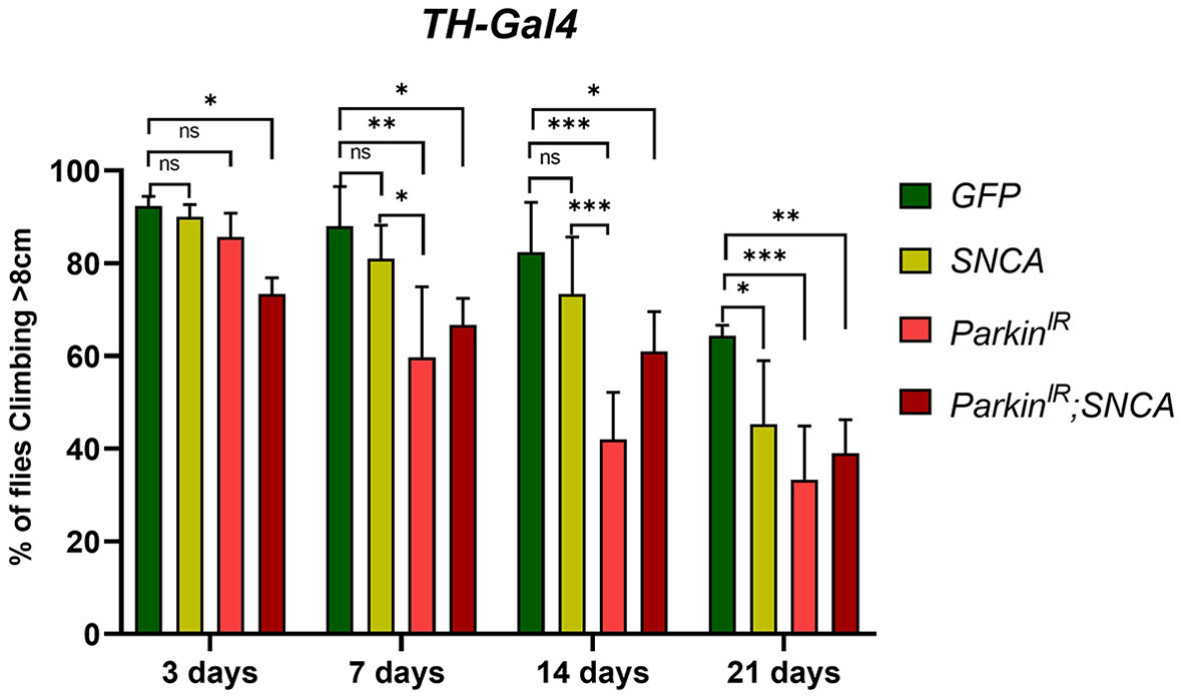
*SNCA overexpression* and *parkin* downregulation (*parkin*^*IR*^) independently, and together (*parkin*^*IR*^*; SNCA*) in dopaminergic neurons (DA), exhibit locomotor dysfunctions: Climbing assay indicates loss of locomotor function with age in flies. A total of 30 (*N* = 30) flies were used per genotype and 10 flies (*n* = 10) were used for the climbing assay. Data is represented as mean with SEM. Statistical analysis was performed using Two-way ANOVA followed by Tukey's multiple comparison test. *P* value: ^*^(0.033), ^**^(0.002), ^***^(< 0.001), ns-not significant (0.12).

The α-synuclein-induced neurotoxicity (i.e., survival, locomotor defect, and DA neuronal death) is restored by *parkin* in *in-vitro* and *in-vivo* models (Petrucelli et al., 2002; Oluwatosin-Chigbu et al., 2003; Yang et al., 2003; Khandelwal et al., 2010). Hence, to test whether parkin is involved in α-synuclein mediated PD condition, we created double transgenes with RNAi of *parkin* (*parkin*^*IR*^ here onward) and *UAS-SNCA* and expressed in DA neurons using *TH-Gal4*. Using H3C (DSHB-S1-890) antibody we confirmed the expression of α-synuclein in DA neurons of *UAS-SNCA* (Supplementary Figure 1A) and double transgenes through fluorescence microscopy (Supplementary Figure 1B) and downregulation of *parkin* through real-time PCR (**Figures 3A**, **B**, 7-day and 21-day respectively). We observed that flies that expressed *SNCA* with *parkin* knockdown (*UAS-parkin*^*IR*^; *UAS-SNCA*) displayed loss of climbing ability as compared to control (Figure 1); however, this was to a lesser extent with respect to *parkin* downregulation alone in age an age-dependent manner. Though, both *SNCA* overexpression and *parkin* downregulation independently displayed a loss of climbing ability with age (3–21 days) as compared to control (*TH*>*GFP*) (Figure 1), supporting the existing studies (Feany and Bender, 2000; Wang et al., 2007; Paricio and Muñoz-Soriano, 2011; Zhang et al., 2013; Mohite et al., 2018). We also confirmed the phenotype of *parkin* downregulation by overexpressing *wild type-parkin* with *SNCA* (*UAS-SNCA;UAS-parkin*). Notably, wild-type *parkin* overexpression with *SNCA* was able to restore the climbing ability (Supplementary Figure 2). We also confirmed the overexpression of α-synuclein using H3C antibody in *UAS-SNCA; UAS-parkin* transgene through fluorescence microscopy (Supplementary Figure 1C). These observations suggest that *SNCA* and *parkin* alteration together do not worsen the locomotor defects.

### *SNCA* and *parkin* alterations cause dopaminergic neurodegeneration

**Figure 2:**
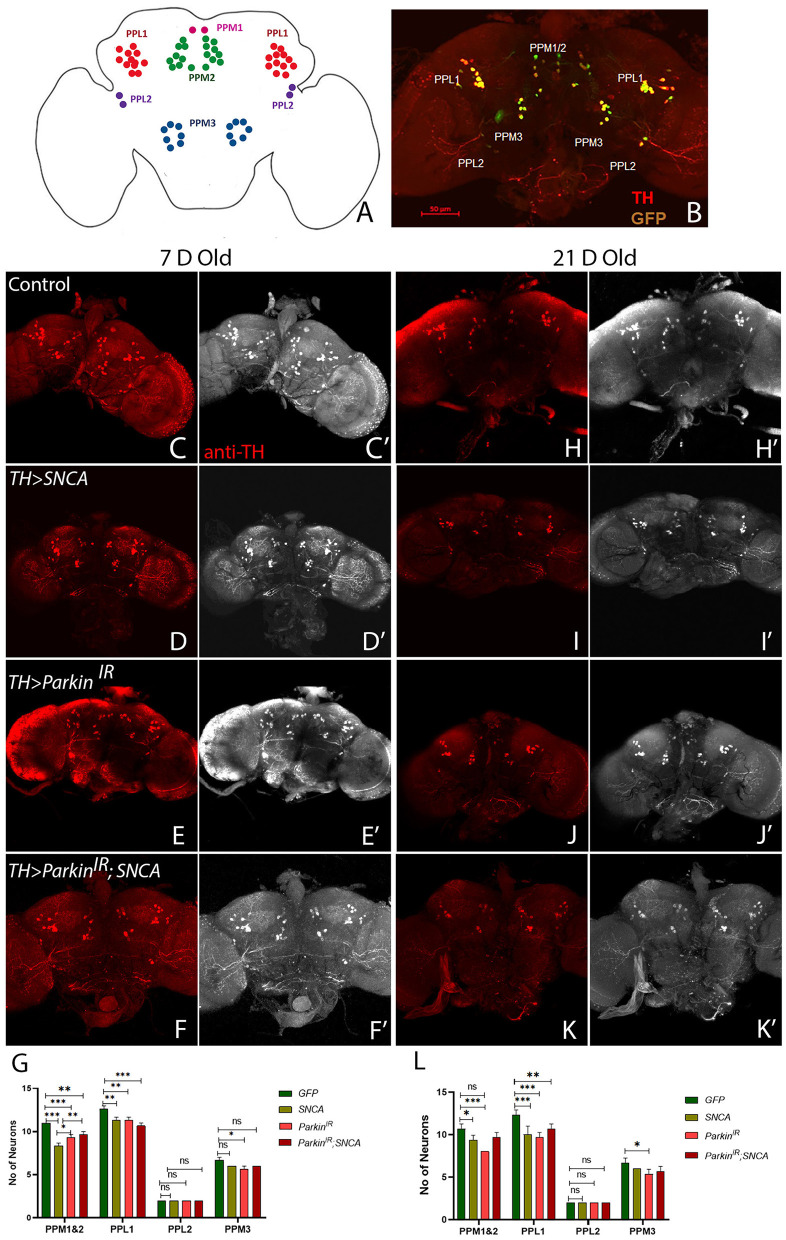
*SNCA* and *parkin*^*IR*^ expression independently and together (*parkin*^*IR*^*; SNCA*) cause DA cluster specific neuronal loss **(A)** Schematic representation of DA neuronal clusters (PPL1, PPM1&2, PPM3, PPL2) in the posterior region of adult brain. **(B)** Representative confocal Maximum Intensity Projection (MIP) of WT adult brain stained with GFP (green) and Tyrosine Hydroxylase (TH) (Red) to reveal DA neurons in the posterior region. An adult brain of the desired genotype was dissected and stained for TH. **(C–G)** 7-day-old adult fly brains show TH stain. **(C, C')** Control flies, **(D, D')** *SNCA* overexpression, **(E, E')** *Parkin*^*IR*^ expression, **(F, F')** *Parkin*^*IR*^*; SNCA* expression, show the TH-stain which is **(G)** quantified. **(H-L)** 21-day-old adult fly brains showing TH stain. **(H, H')** Control flies, **(I, I')** *SNCA* overexpression, **(J, J')** *Parkin*^*IR*^ expression, **(K, K')** *Parkin*^*IR*^*; SNCA* expression shows the TH-stain and is **(L)** quantified. Scale bar 50μm. A total of four adult brains were used (*n* = 4) per genotype. Data is represented as mean with SEM. Statistical analysis was performed using Two-way ANOVA followed by Tukey's multiple comparison test. *P* value: *(0.033), **(0.002), ***(< 0.001), ns-not significant (0.12).

In PD, cardinal motor symptoms are caused by the death of DA neurons in the substantia nigra pars compacta (SNpc). Thus, we examined the DA neuronal clusters in adult fly brains using antibodies against tyrosine hydroxylase to investigate whether locomotor defects observed in *SNCA* and *parkin* alteration are due to loss of DA neurons. We have explored posterior protocerebrum DA neuronal clusters: PPM1/2, PPM3, PPL1, and PPL2 (Figure 2A). To visualize the DA neurons in the adult brain, we stained the DA neurons of the *TH-Gal4* driving *GFP* flies with TH antibody (Figure 2B), hence confirming the activity of *TH-Gal4*>*UAS-GFP* and TH antibody. In our study, only TH-positive neurons were monitored for DA neuron quantification. We have found a substantial reduction in specific DA neuronal clusters in *UAS-SNCA, UAS-parkin*^*IR*^ individually, and the *UAS-parkin*^*IR*^; *UAS-SNCA* transgene adult brain with respect to age-matched controls (Figure 2). In 7-day-old adult fly brains, quantification of PPL1, PPM1&2, PPM3, and PPL2 DA neuron clusters, *SNCA*, and *parkin*^*IR*^ showed significant reduction in number of DA neurons only in PPL1 and PPM1&2 clusters (Figures 2C–E', G). *UAS-parkin*^*IR*^; *UAS-SNCA* also showed a significant reduction in the number of DA neurons in PPL1 and PPM1&2 as compared to control (Figures 2F, F', G). However, the number of DA neuronal losses in *UAS-parkin*^*IR*^; *UAS-SNCA* adult fly brains were less pronounced as compared to *SNCA* and *parkin*^*IR*^ alone (Figures 2D–F', G). In 21-day-old fly brains, DA neurons numbers were further decreased in PPL1 and PPM1 & 2 clusters in a similar manner to 7-day-old adult fly brains of *SNCA, parkin*^*IR*^, and in *UAS-parkin*^*IR*^; *UAS-SNCA* (Figures 2H–K', L). In PPM3 DA neuron clusters, we have observed that the numbers of DA neurons were reduced only in *parkin*^*IR*^ adult fly brains (Figures 2G, L). In the PPL2 DA cluster, we have observed no change in the number of DA neurons in *UAS-SNCA, UAS-parkin*^*IR*^, and *UAS-parkin*^*IR*^; *UAS-SNCA* of 7-day-old and 21-day-old adult fly brains. These observations suggest that *SNCA* and *parkin* cause DA clusters specific neuronal loss in the posterior region of adult fly brain.

### *SNCA* affects the expression of *parkin* at the transcriptional level but not at the translational level

**Figure 3:**
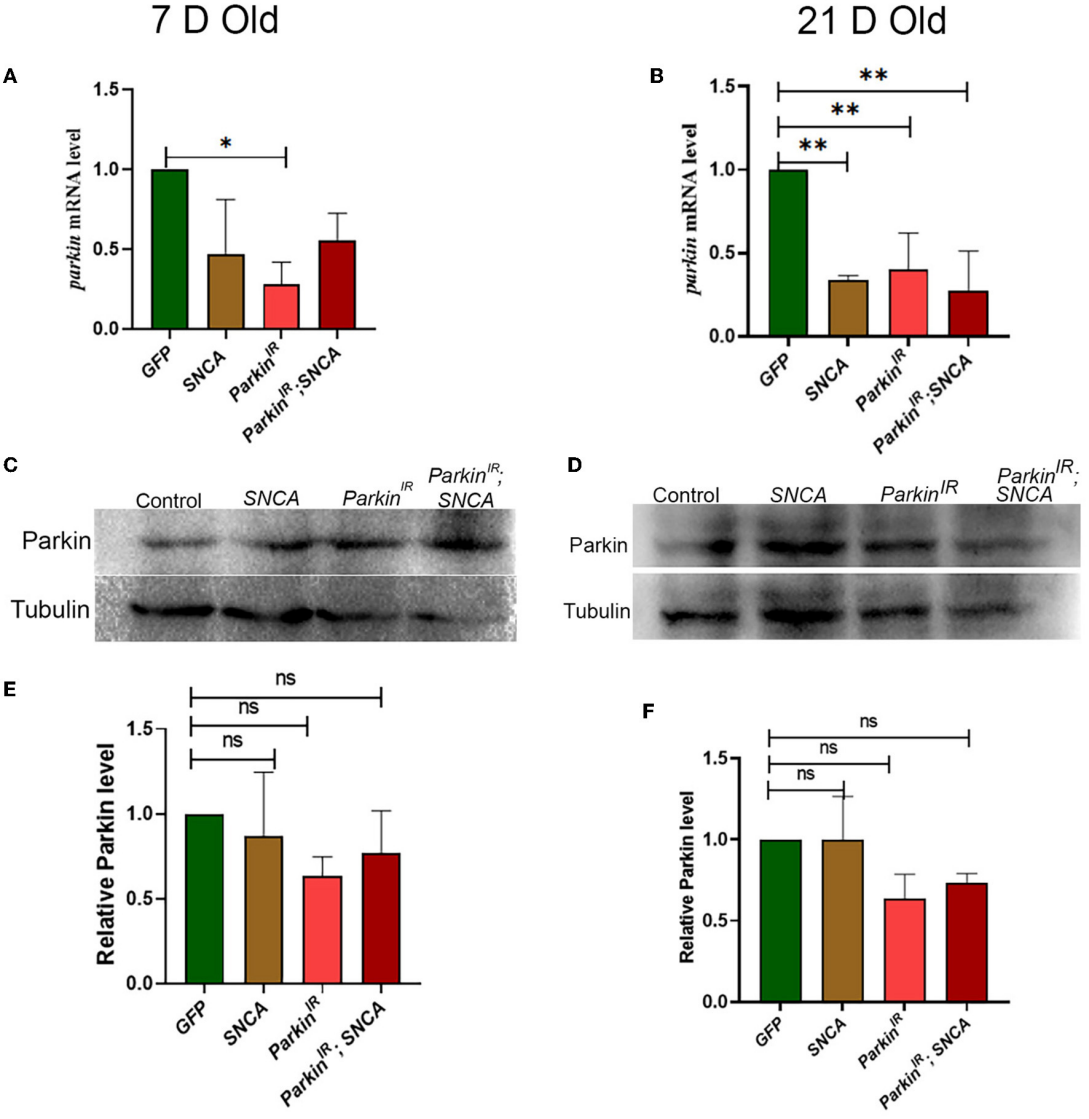
*SNCA* affects the expression of *parkin* at the transcriptional level but not the translational level. **(A)** 7-day old *parkin* mRNA level **(B)** 21-day-old *parkin* mRNA level. **(C)** Parkin immunoblot and **(E)** quantification of 7-day-old adult brains normalized to beta-Tubulin. **(D)** Parkin immunoblot and **(F)** quantification of 21-day old adult brains normalized to beta-Tubulin. Data is represented as mean with SEM. Statistical analysis was performed using One-way ANOVA followed by Tukey's multiple comparison test. *P* value: *(0.033), ** (0.002), ns-not significant (0.12).

Several *in-vitro* and *in-vivo* studies have shown the reduced neurotoxicity caused by *SNCA* upon *parkin* overexpression and suggested that parkin plays a vital role in the molecular pathway of PD pathogenesis. It has been reported that mutations in *SNCA* and *parkin* affect DA neuronal loss as well as the formation of Lewy bodies (Madsen et al., 2021). However, the effect of wild-type *SNCA* on *parkin* is not much explored. Therefore, we tested the *parkin* mRNA and protein levels of flies with *UAS-parkin*^*IR*^; *UAS-SNCA, SNCA* overexpression, and *parkin* downregulation independently. We found decreased *parkin* mRNA levels in *UAS-parkin*^*IR*^; *UAS-SNCA* flies as compared to control flies; however, this decreased transcript was at a lesser extent with respect to *parkin downregulation* and *SNCA* overexpression independently in 7-day adult fly brains (Figure 3A). In 21-day-old adult fly brains with *UAS-parkin*^*IR*^; *UAS-SNCA* has not shown a further decrease in *parkin* mRNA level as compared to control and as well as with *SNCA* overexpression and *parkin* downregulation independently (Figure 3B). Wilkaniec et al., 2019 have reported a decreased parkin protein level upon α-synuclein oligomerization which induces cell death in an *in-vitro* model. In contrast, in our study, we have found no significant change in parkin protein level upon *SNCA* overexpression in 7 (Figures 3C, E) and 21-day-old (Figures 3D, F) adult fly brains. However, we observed decreased parkin protein levels in flies expressing *UAS-parkin*^*IR*^; *UAS-SNCA* as compared to control but it was to a lesser extent to *parkin* downregulation independently (Figures 3E, F). Altogether, these data suggest that *SNCA* affects the *parkin* level.

### *SNCA* and *parkin* alteration affect mitochondrial morphology independently in PPL1 and PPM3 clusters of the adult fly brain

**Figure 4:**
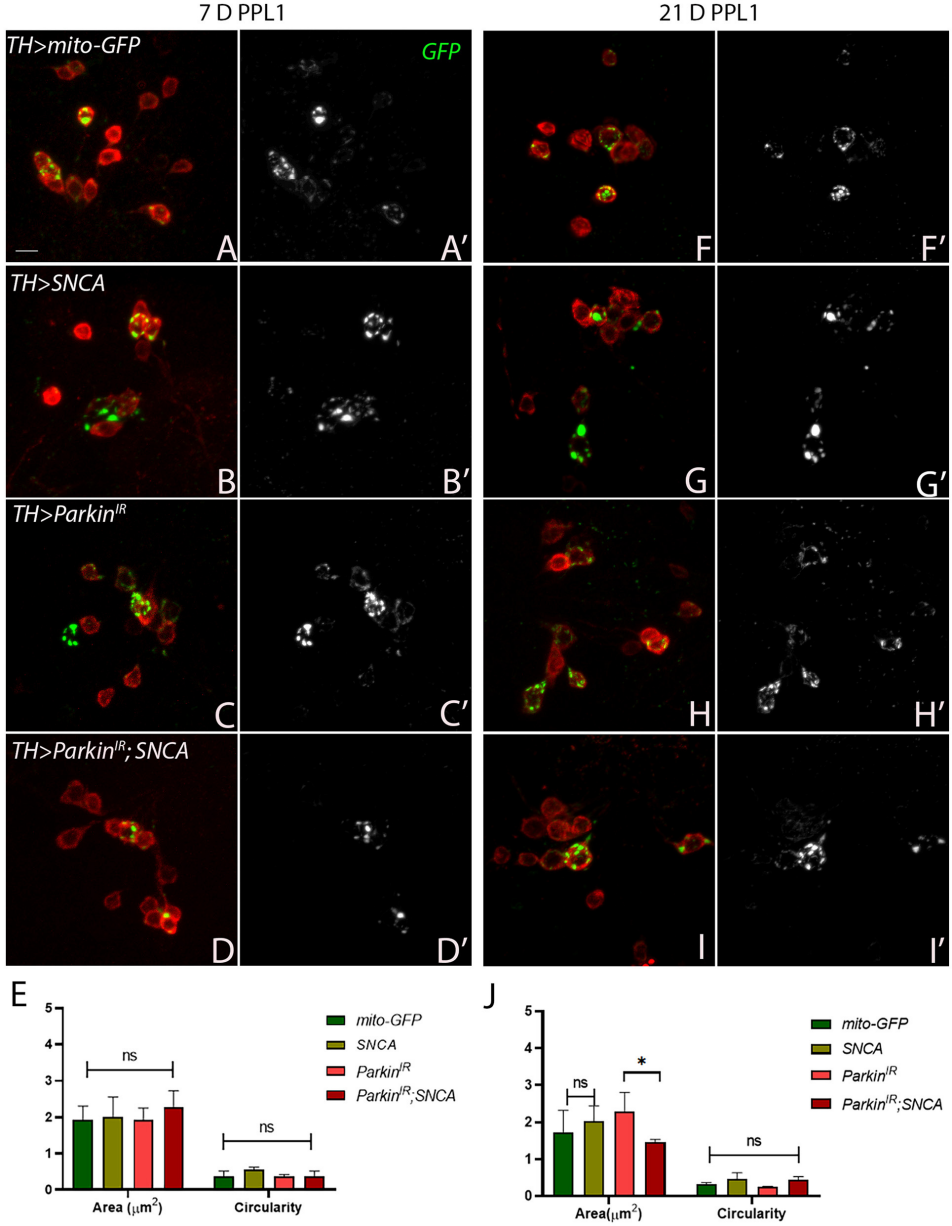
*SNCA* overexpression results in swollen mitochondria, *parkin*^*IR*^ expression has shown elongated whereas together (*parkin*^*IR*^*; SNCA*) shows fragmented mitochondria in PPL1 DA clusters. Adult brains of the desired genotype expressing the mitochondria-targeted green fluorescent protein (mitoGFP) in TH- TH-positive (red) cells. **(A–J)** 7-day & 21-day old adult fly brains showing mitoGFP in PPL1 cluster. Control brains showing mitoGFP at **(A, A')** 7-day and **(F, F')** 21-day. *SNCA* overexpressing flies show mitoGFP **(B, B')** in 7-day and in **(G, G')** 21-day. *Parkin*^*IR*^-expressing flies show mitoGFP in **(C, C')** 7-day and in **(H, H')** 21-day. *Parkin*^*IR*^*; SNCA* expressing flies show mitoGFP in **(D, D')** 7-day and further enhanced in **(I, I')** 21-day. **(E)** Quantification of mitochondria morphology (area and circularity) was done using ImageJ Mito-Morphology Macro. Scale bar 10μm. A total of four adult brains were used (*n* = 4) per genotype. Data is represented as mean with SEM. Statistical analysis was performed using Two-way ANOVA followed by Tukey's multiple comparison test. *P* value: *(0.033), ns-not significant (0.12).

Mitochondria are highly dynamic organelles and maintenance of mitochondrial morphology is essential for the survival of the neurons. Therefore, we investigated whether α-synuclein and parkin alteration-induced PD phenotypes have any relation with mitochondrial morphology. We have considered only PPL1 and PPM3 DA neuronal clusters for mitochondrial morphology assessment. This is because we have found a decrease in the number of TH-positive DA neurons in PPL1 clusters of *UAS-parkin*^*IR*^; *UAS-SNCA* (Figure 2), *SNCA* overexpression, and *parkin* downregulation independently and in PPM3 due to *parkin* downregulation only. We assessed the mitochondrial morphology using *UAS-mitoGFP* in TH-positive neurons. We have found that in PPL1 neuronal clusters, *UAS-parkin*^*IR*^; *UAS-SNCA* (Figures 4D, D', E; Supplementary Figure 4D) has shown swollen mitochondria as compared to control (*TH*>*mito-GFP*) (Figures 4A, A'; Supplementary Figure 4A) in 7-day adult fly brains. Whereas we observed fragmented mitochondria in 21-day-old adult fly brains (Figures 4I, I', J; Supplementary Figure 4H) as compared to control (Figures 4F, F', J; Supplementary Figure 4E). *SNCA* overexpression (Figures 4B, B', E; Supplementary Figure 4B) and *parkin* downregulation (Figures 4C, C', E; Supplementary Figure 4C) independently have shown swollen and/or enlarged mitochondria, which degenerate, in 7-day-old adult fly brains, as compared to control (Figures 4A, A', E; Supplementary Figure 4A). *SNCA* overexpression (Figures 4G, G', J; Supplementary Figure 4F) and *parkin* downregulation (Figures 4H, H', J; Supplementary Figure 4G) independently have shown further enhanced mitochondrial morphology in 21-day-old adult fly brains as compared to control (Figures 4F, F', J; Supplementary Figure 4E).

**Figure 5:**
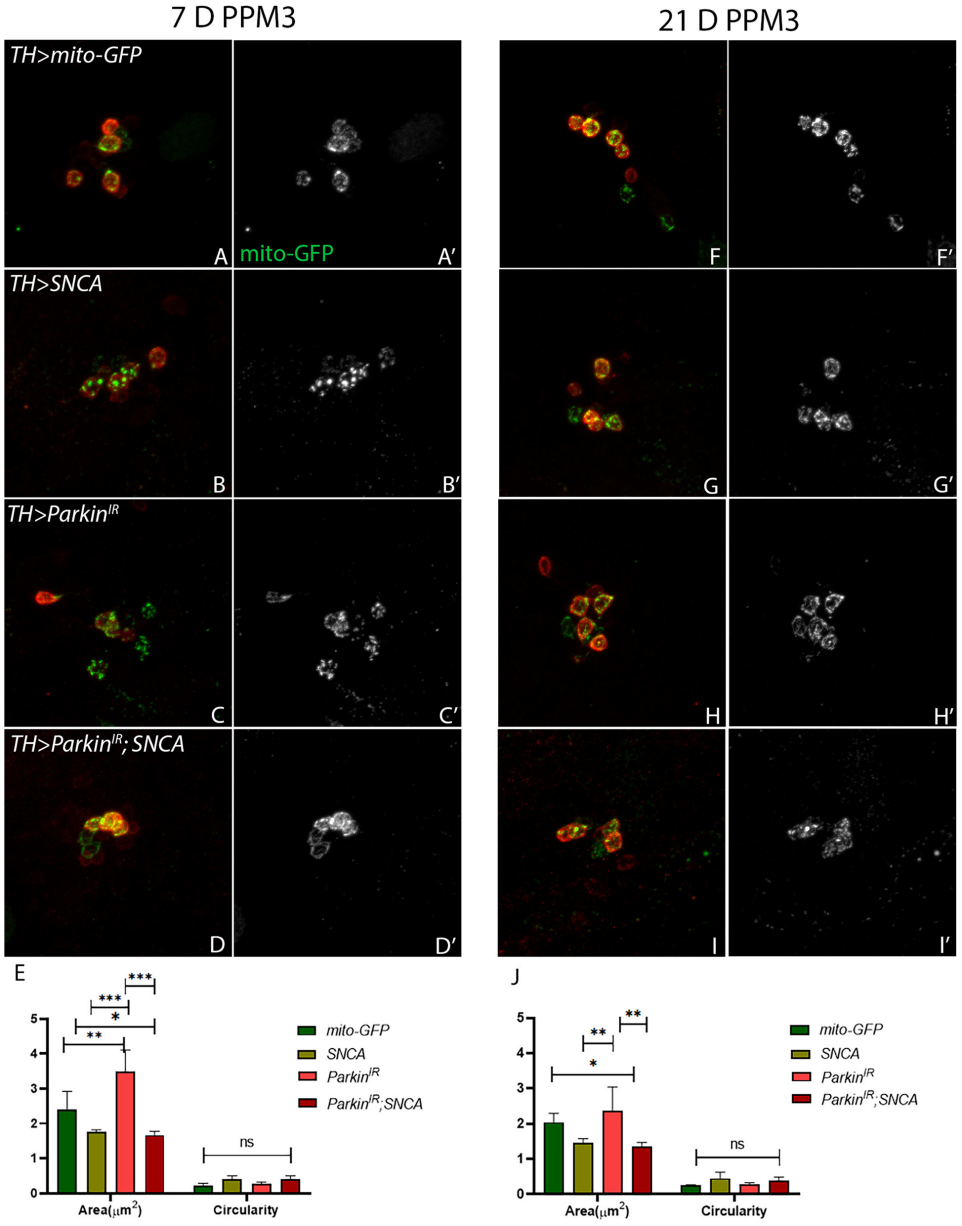
*SNCA* overexpression and *parkin*^*IR*^*; SNCA* have shown fragmented mitochondria, whereas *parkin*^*IR*^ expression has shown elongated mitochondria in PPM3 DA clusters. Adult brains of the desired genotype expressing the mitochondria-targeted green fluorescent protein (mitoGFP) in TH- TH-positive (red) cells. **(A–J)** 7-day & 21-day old adult fly brains showing mitoGFP in PPM3 cluster. Control brains showing mitoGFP at **(A, A')** 7-day and **(F, F')** 21-day. *SNCA* overexpressing flies show mitoGFP **(B, B')** in 7-day and in **(G, G')** 21-day. *Parkin*^*IR*^ expressing flies show mitoGFP in **(C, C')** 7-day and in **(H, H')** 21-day. *Parkin*^*IR*^*; SNCA* expressing flies show mitoGFP in **(D, D')** 7-day and in **(I, I')** 21-day also. **(E)** Quantification of mitochondria morphology (area and circularity) was done using ImageJ Mito-Morphology Macro. Scale bar 10 μm. A total of four adult brains were used (*n* = 4) per genotype. Data is represented as mean with SEM. Statistical analysis was performed using Two-way ANOVA followed by Tukey's multiple comparison test. *P* value: *(0.033), **(0.002), ***(< 0.001), ns-not significant (0.12).

In PPM3 neuronal clusters, *UAS-parkin*^*IR*^; *UAS-SNCA* (Figures 5D, D', E) and *SNCA* overexpression (Figures 5B, B', E) have shown fragmented mitochondria in 7-day-old adult fly brains as compared to control (Figures 5A, A', E). In 21-day-old adult brains of *UAS-parkin*^*IR*^; *UAS-SNCA* (Figures 5I, I', J) and *SNCA* overexpression (Figures 5G, G', J) have also shown fragmented mitochondria as compared to control (Figures 5F, F', J) which do not degenerate. The *parkin* downregulation only has shown enlarged and/or swollen mitochondria in PPM3 clusters which degenerate, in 7-day (Figures 5C, C', 5E) as well as in 21-day old adult fly brains (Figures 5H, H', J) as compared to control (Figures 5A, A', E). To confirm the role of parkin in *SNCA*-induced mitochondrial morphology defects, we performed mitochondrial fractionation. We observed non-statistically different reductions in mitochondrial localization of parkin protein (Supplementary Figure 3). Hence, these data confirm the role of parkin being independent of α-synuclein to cause altered mitochondrial morphology in PD progression.

## Discussion

Here, we characterize the cluster-specific DA neuronal loss associated with the interaction of *SNCA* and *parkin*. We have highlighted the effect of *SNCA* overexpression and *parkin* knockdown together in terms of DA neuronal loss in PPL1 and PPM3 clusters of adult fly brains over a time period of 21 days.

*SNCA* and *parkin* mutations have been found to be involved in motor (postural instability, tremor, bradykinesia) and non-motor symptoms (sleep disorders, depression, anxiety, hallucinations) in PD patients. *SNCA* overexpression and *parkin* mutation have also been shown to cause age-dependent locomotor dysfunction and neurodegeneration in *in-vivo* models (Feany and Bender, 2000; Wang et al., 2007; Ordonez et al., 2018; Yan et al., 2019). In our study, *SNCA* overexpression and *parkin* knockdown in DA neurons have also shown progressive locomotor dysfunction. Interaction of *SNCA* and *parkin* in PPL1 clusters results in progressive locomotor dysfunctions. However, the effect of genetic alterations is not as pronounced to indicate an additive effect. This is further supported by observed mitochondrial morphology in *UAS-parkin*^*IR*^; *UAS-SNCA*. With respect to the reports published previously, our work uncovers the relationship between *SNCA* and *parkin* in an *in-vivo* system in a novel way by bringing the gene alterations together. This enables us to understand the relationship, between *SNCA* and *parkin* at both transcriptional and translational levels.

Studies have reported that wild-type *SNCA* causes a reduction in the number of TH-positive neurons in PPM1&2 and PPL1 clusters but not in PPM3 clusters (Trinh et al., 2008, 2010; Barone et al., 2011; Agostini et al., 2023). We have also found that *SNCA* overexpression has reduced the number of TH-positive neurons in PPM1&2 and PPL1 and not in PPM3, which are consistent with aforementioned reports. Flies with *parkin* mutation have also been shown to cause progressive loss of DA neurons in the PPL1 cluster but not in PPM3 (Whitworth et al., 2005; Wang et al., 2007) after 20 days post-eclosion. No loss of neurons has been reported in the dorsomedial clusters (DMC) (also known as PPM) in *parkin* loss of function mutation in the adult fly brain after 21 days post-eclosion (Pesah et al., 2004). However, in our study, parkin knockdown alone shows loss of DA neurons in PPM3 along with PPM1&2 and PPL1 clusters in 7 and 21-day-old adult fly brains. *SNCA* with *parkin* knockdown (*UAS-parkin*^*IR*^; *UAS-SNCA*) showed a decreased number of DA neurons in PPM1&2, PPL1 clusters as compared to control, though at a lesser extent with *SNCA* and *parkin* knockdown independently. Numbers of DA neurons in PPL2 clusters were unaltered in *SNCA* and *parkin* knockdown independently and together, in 7-day as well as in 21-day-old fly brains. Hence, these observations suggest that DA neuronal loss was correlated with locomotor dysfunctions. Since we did not observe aggravated phenotype in *SNCA* overexpression and *parkin* knockdown together (*UAS-parkin*^*IR*^; *UAS-SNCA)*; this may suggest that *SNCA* doesn't affect *parkin* directly. Alternatively, this could also mean that *parkin* downregulation is not the only mechanism involved in *SNCA*-induced locomotor dysfunction and neurodegeneration.

Neurons have highly dynamic energy requirements, and hence intact mitochondrial morphology is an important aspect to preserve neuronal health. In post-mortem brains of Parkinson's patients, it has been shown that α-synuclein localizes to mitochondria and affects mitochondrial homeostasis (Devi et al., 2008; Nakamura et al., 2011; Wang et al., 2019; Choi et al., 2022). Although, α-synuclein does not have an exact mitochondrial targeting sequence, studies suggest that α-synuclein contains a cryptic mitochondrial targeting sequence in the N-terminus region (Devi et al., 2008). Recently, it has been reported that N-terminus of α-synuclein plays a role in mitochondrial fragmentation via a DRP1-dependent pathway in *Drosophila* (Krzystek et al., 2021). In *Drosophila, C. elegans*, dorsal root ganglia of *Danio rerio* (zebra fish) and in cellular models as well, *SNCA* overexpression causes mitochondrial fragmentation (Kamp et al., 2010; Butler et al., 2012; O'Donnell et al., 2014). In our study, *SNCA* overexpression has caused more elongated/or swollen mitochondria in PPL1 clusters (Supplementary Figures 4B, F), while in PPM3 clusters, it results in more fragmented mitochondria in a progressive manner. These results are thus align with the DA neuronal loss in PPL1 clusters but not in PPM3 clusters. This may also be an indication of some other mechanisms involved in rescuing the effect of *SNCA* overexpression and *parkin* knockdown together.

Loss-of-function mutations in *parkin* are the most prevalent cause of recessive form PD (Corti et al., 2011). Upon mitochondria depolarization, parkin is activated by PINK1 and promotes degradation of Mitofusin 1 and 2 (Poole et al., 2008; Gegg et al., 2010; Sarraf et al., 2013) and recruits Drp1 to mitochondria which leads to fission (Buhlman et al., 2014). Parkin is also involved in the selective degradation of damaged mitochondria through the mitophagy process (Pickrell and Youle, 2015). In tissues of *parkin*-null *Drosophila* mutants, swollen mitochondria have been observed and this suggests that *parkin* may either promote fission or inhibit fusion (Greene et al., 2003; Pesah et al., 2004). Conversely, in DA neurons of *parkin* knockout mice, more fragmented mitochondria have been shown to cause neuronal loss (Noda et al., 2020). The presence of *parkin* mutation in causing accumulation of dysfunctional mitochondria in PD patients has also been established. In our study, *parkin* downregulation caused the enlargement of mitochondria in PPL1 (Supplementary Figures 4C, G) and PPM3 clusters in age age-dependent manner. These results were correlating with the DA neuronal loss in PPL1 and PPM3 clusters.

Several studies have reported that overexpression of *parkin* restores mitochondrial morphology and function caused by *SNCA*, but it is still not clear whether this is through a direct link between *parkin* and *SNCA*, or the neuroprotective role of parkin in maintaining mitochondrial dynamics (Kamp et al., 2010; Lonskaya et al., 2013; Krzystek et al., 2021). In the *in-vitro* model, exogenous α-synuclein oligomers or fibrils caused a reduction in *parkin* expression and wild-type *parkin* overexpression rescues α-synuclein-induced mitochondrial fragmentation (Wilkaniec et al., 2021). However, they have shown that the toxic effects of α-synuclein on mitochondria were higher as compared to *parkin* silencing-induced mitochondrial dysfunction and suggested that α-synuclein-induced *parkin* downregulation is not the only mechanism for mitochondrial dysfunction (Wilkaniec et al., 2021). Similarly, in our study, overexpression of *SNCA* with *parkin* downregulation (*UAS-parkin*^*IR*^; *UAS-SNCA*) shows more fragmented mitochondria in PPL1 (Supplementary Figure 4E) as well in PPM3 clusters in age-dependent manner, which is just opposite to *SNCA* overexpression and *parkin* downregulation individually. We have found no significant changes in parkin expression at the protein level in *SNCA* overexpressed flies; however, *parkin* transcript was significantly reduced. In our fractionation studies as well, we did not see statistically significant differences in the localization of Parkin protein. This warrants that further studies need to be carried out to validate the transcriptional correlation. Also, critical differences in gene expression related to mitochondrial morphology can be explored further. It is important to note that our observations of mitochondrial morphology in specific clusters pertain to the TH-positive neurons only, based on the limited number of trials performed. Thus, further comprehension of mitochondrial morphology within these particular clusters can be attained by exploring other aspects of mitochondrial homeostasis, such as mitochondrial transport and mitophagy, in the progression of Parkinson's Disease.

**Figure 6:**
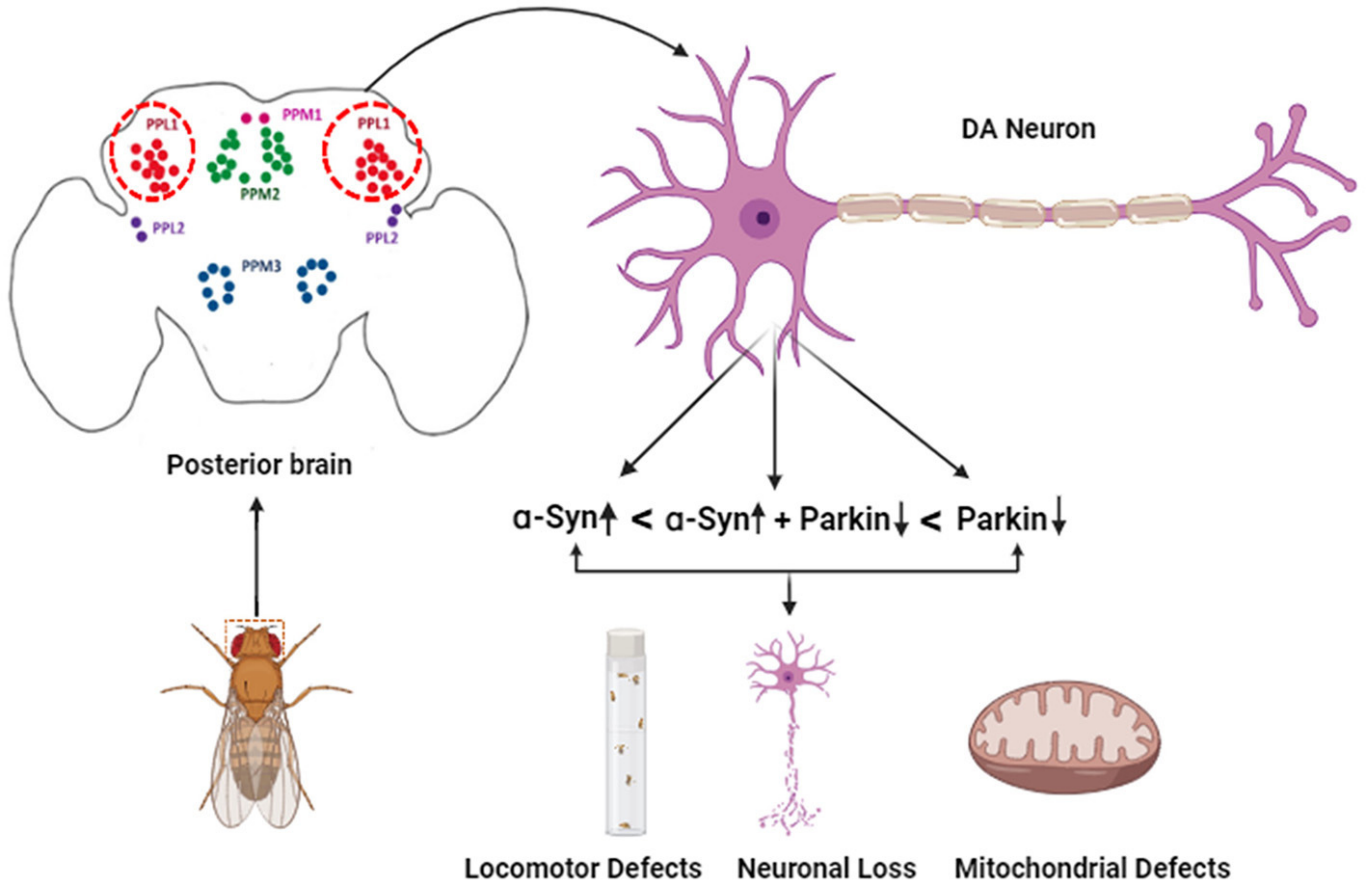
Schematic representation of the effect of α-syn and parkin on specific DA neuronal clusters in the adult fly brain.

The current study provides insights into cellular and molecular etiology in the case of PD in a time-dependent manner specifically in DA neurons, using an overexpression system. Since neuronal mitochondria are highly dynamic, depending on ever-changing metabolic requirements, the morphology changes driving degeneration are limited to TH-positive neurons (Figure 6). Using cell isolation techniques from individual DA neuronal clusters of brains will be able to provide more insights at the individual neuronal and organelle level since the number of these neurons is limited. Furthermore, to understand the mechanisms involved in regulation at the organelle level, more research will be required in other animals to understand if these interactions are conserved at the cellular and molecular level affecting the pathogenesis of Parkinson's disease. In addition, it would be crucial to understand if mitochondria are affected by other direct or indirect genetic and molecular factors affecting the progression of Parkinson's disease.

## Data availability statement

The original contributions presented in the study are included in the article/Supplementary material, further inquiries can be directed to the corresponding authors.

## Author contributions

SN: Conceptualization, Data curation, Formal analysis, Investigation, Resources, Visualization, Writing – original draft. AS: Conceptualization, Writing – review & editing. MT: Conceptualization, Writing – review & editing, Project administration, Writing – original draft.

## References

[B1] Agostini F. Bubacco L. Chakrabarti S. Bisaglia M. (2023). α-synuclein toxicity in *drosophila melanogaster* is enhanced by the presence of iron: implications for Parkinson's disease. Antioxidants 12, 261. 10.3390/antiox12020261 36829820 PMC9952566

[B2] Appel-Cresswell S. Vilarino-Guell C. Encarnacion M. Sherman H. Yu I. Shah B.. (2013). Alpha-synuclein p.H50Q, a novel pathogenic mutation for Parkinson's disease. Mov. Disord. 28, 811–813. 10.1002/mds.25421 23457019

[B3] Aryal B. Lee Y. (2019). Disease model organism for Parkinson disease: *Drosophila melanogaster*. BMB Rep. 52, 250–258. 10.5483/BMBRep.2019.52.4.204 30545438 PMC6507844

[B4] Barone M. C. Sykiotis G. P. Bohmann D. (2011). Genetic activation of Nrf2 signaling is sufficient to ameliorate neurodegenerative phenotypes in a Drosophila model of Parkinson's disease. DMM Dis. Model. Mech. 4, 701–707. 10.1242/dmm.007575 21719443 PMC3180234

[B5] Bier E. (2005). Drosophila, the golden bug, emerges as a tool for human genetics. Nat. Rev. Genet. 6, 9–23. 10.1038/nrg1503 15630418

[B6] Brand a H. Perrimon N. (1993). Ature. Development 118, 401–415. 10.1242/dev.118.2.401 8223268

[B7] Buhlman L. Damiano M. Bertolin G. Ferrando-Miguel R. Lombès A. Brice A.. (2014). Functional interplay between Parkin and Drp1 in mitochondrial fission and clearance. Biochim. Biophys. Acta - Mol. Cell Res. 1843, 2012–2026. 10.1016/j.bbamcr.2014.05.012 24878071

[B8] Burré J. Sharma M. Tsetsenis T. Buchman V. Etherton M. R. Südhof T. C. (2010). α-Synuclein promotes SNARE-complex assembly *in vivo* and *in vitro*. Science 329, 1663–1667. 10.1126/science.1195227 20798282 PMC3235365

[B9] Butler E. K. Voigt A. Lutz A. K. Toegel J. P. Gerhardt E. Karsten P.. (2012). The mitochondrial chaperone protein TRAP1 mitigates α-synuclein toxicity. PLoS Genet. 8. 10.1371/journal.pgen.1002488 22319455 PMC3271059

[B10] Cackovic J. Gutierrez-Luke S. Call G. B. Juba A. O'Brien S. Jun C. H.. (2018). Vulnerable parkin loss-of-function Drosophila dopaminergic neurons have advanced mitochondrial aging, mitochondrial network loss and transiently reduced autophagosome recruitment. Front. Cell. Neurosci. 12, 1–14. 10.3389/fncel.2018.00039 29497364 PMC5818410

[B11] Chartier S. Duyckaerts C. (2018). Is Lewy pathology in the human nervous system chiefly an indicator of neuronal protection or of toxicity? Cell Tissue Res. 373, 149–160. 10.1007/s00441-018-2854-6 29869713

[B12] Choi M. L. Chappard A. Singh B. P. Maclachlan C. Rodrigues M. Fedotova E. I.. (2022). Pathological structural conversion of α-synuclein at the mitochondria induces neuronal toxicity. Nat. Neurosci. 25, 1134–1148. 10.1038/s41593-022-01140-3 36042314 PMC9448679

[B13] Corti O. Lesage S. Brice A. (2011). What genetics tells us about the causes and mechanisms of Parkinson's disease. Physiol. Rev. 91, 1161–1218. 10.1152/physrev.00022.2010 22013209

[B14] Dagda R. K. Cherra S. J. Kulich S. M. Tandon A. Park D. Chu C. T. (2009). Loss of PINK1 function promotes mitophagy through effects on oxidative stress and mitochondrial fission. J. Biol. Chem. 284, 13843–13855. 10.1074/jbc.M808515200 19279012 PMC2679485

[B15] Dawson T. M. Ko H. S. Dawson V. L. (2010). Genetic animal models of Parkinson's disease. Neuron 66, 646–61. 10.1016/j.neuron.2010.04.034 20547124 PMC2917798

[B16] Deng H. Dodson M. W. Huang H. Guo M. (2008). The Parkinson's disease genes pink1 and parkin promote mitochondrial fission and/or inhibit fusion in Drosophila. Proc. Natl. Acad. Sci. U. S. A. 105, 14503–14508. 10.1073/pnas.0803998105 18799731 PMC2567186

[B17] Devi L. Raghavendran V. Prabhu B. M. Avadhani N. G. Anandatheerthavarada H. K. (2008). Mitochondrial import and accumulation of α-synuclein impair complex I in human dopaminergic neuronal cultures and Parkinson's disease brain. J. Biol. Chem. 283, 9089–9100. 10.1074/jbc.M710012200 18245082 PMC2431021

[B18] Feany M. B. Bender W. W. (2000). A Drosophila model of Parkinson's disease. Nature 404, 394–8. 10.1038/35006074 10746727

[B19] Gegg M. E. Cooper J. M. Chau K. Y. Rojo M. Schapira A. H. V. Taanman J. W. (2010). Mitofusin 1 and mitofusin 2 are ubiquitinated in a PINK1/parkin-dependent manner upon induction of mitophagy. Hum. Mol. Genet. 19, 4861–4870. 10.1093/hmg/ddq419 20871098 PMC3583518

[B20] Gogia N. Sarkar A. Singh A. (2017). An undergraduate cell biology lab: western blotting to detect proteins from Drosophila eye. Drosophila Inform. Serv. 100, 218–225. Available online at: https://www.ou.edu/journals/dis/DIS100/DIS%20100.pdf

[B21] Goldberg M. S. Fleming S. M. Palacino J. J. Cepeda C. Lam H. A. Bhatnagar A.. (2003). Parkin-deficient mice exhibit nigrostriatal deficits but not loss of dopaminergic neurons. J. Biol. Chem. 278, 43628–43635. 10.1074/jbc.M308947200 12930822

[B22] Greene J. C. Whitworth A. J. Kuo I. Andrews L. A. Feany M. B. Pallanck L. J. (2003). Mitochondrial pathology and apoptotic muscle degeneration in Drosophila parkin mutants. Proc. Natl. Acad. Sci. U. S. A. 100, 4078–4083. 10.1073/pnas.0737556100 12642658 PMC153051

[B23] Haywood A. F. M. Staveley B. E. (2004). Parkin counteracts symptoms in a Drosophila model of Parkinson's disease. BMC Neurosci. 5, 1–12. 10.1186/1471-2202-5-14 15090075 PMC419346

[B24] Heisenberg M. (2003). Mushroom body memoir: from maps to models. Nat. Rev. Neurosci. 4, 266–275. 10.1038/nrn1074 12671643

[B25] Jeśko H. Lenkiewicz A. M. Wilkaniec A. Adamczyk A. (2019). The interplay between parkin and alpha-synuclein; possible implications for the pathogenesis of Parkinson's disease. Acta Neurobiol. Exp. (Wars). 79, 279–289. 10.21307/ane-2019-026 31587020

[B26] Johansen K. K. Torp S. H. Farrer M. J. Gustavsson E. K. Aasly J. O. (2018). A case of Parkinson's disease with no lewy body pathology due to a homozygous exon deletion in parkin. Case Rep. Neurol. Med. 2018, 1–4. 10.1155/2018/6838965 30050705 PMC6046180

[B27] Jost W. H. Reichmann H. (2017). “An essay on the shaking palsy” 200 years old. J. Neural Transm. 124, 899–900. 10.1007/s00702-017-1684-0 28155132

[B28] Kachergus J. Roumier C. Mouroux V. Douay X. Lincoln S. Levecque C. (2004). α-synuclein locus duplication as a cause of familial Parkinson's disease. 07, 1167–1169. 10.1016/S0140-6736(04)17103-1 15451224

[B29] Kamp F. Exner N. Lutz A. K. Wender N. Hegermann J. Brunner B.. (2010). Inhibition of mitochondrial fusion by α-synuclein is rescued by PINK1, Parkin and DJ-1. EMBO J. 29, 3571–3589. 10.1038/emboj.2010.223 20842103 PMC2964170

[B30] Khandelwal P. J. Dumanis S. B. Feng L. R. Maguire-Zeiss K. Rebeck G. Lashuel H. A.. (2010). Parkinson-related parkin reduces α-Synuclein phosphorylation in a gene transfer model. Mol. Neurodegener. 5, 47. 10.1186/1750-1326-5-47 21050448 PMC2987994

[B31] Kitada T. Asakawa S. Hattori N. Matsumine H. Yamamura Y. Minoshima S.. (1998). Mutations in the parkin gene cause autosomal recessive juvenile parkinsonism. Nature 392, 605–8. 10.1038/33416 9560156

[B32] Krzystek T. J. Banerjee R. Thurston L. Huang J. Q. Swinter K. Rahman S. N.. (2021). Differential mitochondrial roles for α-synuclein in DRP1-dependent fission and PINK1/Parkin-mediated oxidation. Cell Death Dis. 12, 1–16. 10.1038/s41419-021-04046-3 34404758 PMC8371151

[B33] Li J. Y. Englund E. Holton J. L. Soulet D. Hagell P. Lees A. J.. (2008). Lewy bodies in grafted neurons in subjects with Parkinson's disease suggest host-to-graft disease propagation. Nat. Med. 14, 501–503. 10.1038/nm1746 18391963

[B34] Lonskaya I. Desforges N. M. Hebron M. L. Moussa C. E. H. (2013). Ubiquitination increases parkin activity to promote autophagic a-synuclein clearance. PLoS ONE 8. 10.1371/journal.pone.0083914 24386307 PMC3873413

[B35] Madsen D. A. Schmidt S. I. Blaabjerg M. Meyer M. (2021). Interaction between parkin and α-synuclein in park2-mediated Parkinson's disease. Cells 10, 1–30. 10.3390/cells10020283 33572534 PMC7911026

[B36] Mao Z. Davis R. L. (2009). Eight different types of dopaminergic neurons innervate the Drosophila mushroom body neuropil: anatomical and physiological heterogeneity. Front. Neural Circuits 3, 1–17. 10.3389/neuro.04.005.2009 19597562 PMC2708966

[B37] Mehta A. S. Luz-Madrigal A. Li J.-L. Tsonis P. A. Singh A. (2019). Comparative transcriptomic analysis and structure prediction of novel Newt proteins. PLoS ONE 14, e0220416. 10.1371/journal.pone.0220416 31419228 PMC6697330

[B38] Mohite G. M. Dwivedi S. Das S. Kumar R. Paluri S. Mehra S.. (2018). Parkinson's disease associated α-synuclein familial mutants promote dopaminergic neuronal death in *Drosophila melanogaster*. ACS Chem. Neurosci. 9, 2628–2638. 10.1021/acschemneuro.8b00107 29906099

[B39] Monastirioti M. (1999). Biogenic amine systems in the fruit fly *Drosophila melanogaster*. Microsc. Res. Tech. 45, 106–121. 10332728 10.1002/(SICI)1097-0029(19990415)45:2<106::AID-JEMT5>3.0.CO;2-3

[B40] Nakamura K. (2013). α-Synuclein and mitochondria: partners in crime? Neurotherapeutics 10, 391–9. 10.1007/s13311-013-0182-9 23512373 PMC3701775

[B41] Nakamura K. Nemani V. M. Azarbal F. Skibinski G. Levy J. M. Egami K.. (2011). Direct membrane association drives mitochondrial fission by the Parkinson's disease-associated protein α-synuclein. J. Biol. Chem. 286, 20710–26. 10.1074/jbc.M110.213538 21489994 PMC3121472

[B42] Nicoletti V. Palermo G. Del Prete E. Mancuso M. Ceravolo R. (2021). Understanding the multiple role of mitochondria in Parkinson's disease and related disorders: lesson from genetics and protein–interaction network. Front. Cell Dev. Biol. 9, 1–20. 10.3389/fcell.2021.636506 33869180 PMC8047151

[B43] Noda S. Sato S. Fukuda T. Tada N. Uchiyama Y. Tanaka K.. (2020). Loss of Parkin contributes to mitochondrial turnover and dopaminergic neuronal loss in aged mice. Neurobiol. Dis. 136, 104717. 10.1016/j.nbd.2019.104717 31846738

[B44] O'Donnell K. C. Lulla A. Stahl M. C. Wheat N. D. Bronstein J. M. Sagasti A. (2014). Axon degeneration and PGC-1α-mediated protection in a zebrafish model of α-synuclein toxicity. DMM Dis. Model. Mech. 7, 571–582. 10.1242/dmm.013185 24626988 PMC4007408

[B45] Olanow C. W. Brundin P. (2013). Parkinson's disease and alpha synuclein: is parkinson's disease a prion-like disorder? Mov. Disord. 28, 31–40. 10.1002/mds.25373 23390095

[B46] Oluwatosin-Chigbu Y. Robbins A. Scott C. W. Arriza J. L. Reid J. D. Zysk J. R. (2003). Parkin suppresses wild-type α-synuclein-induced toxicity in SHSY-5Y cells. Biochem. Biophys. Res. Commun. 309, 679–684. 10.1016/j.bbrc.2003.08.059 12963044

[B47] Ordonez D. G. Lee M. K. Feany M. B. (2018). α-synuclein induces mitochondrial dysfunction through spectrin and the actin cytoskeleton. Neuron 97, 108–124.e6. 10.1016/j.neuron.2017.11.036 29249285 PMC5755717

[B48] Paricio N. Muñoz-Soriano V. (2011). Drosophila models of Parkinson's disease: Discovering relevant pathways and novel therapeutic strategies. Parkinsons. Dis. 2011, 520640. 10.4061/2011/520640 21512585 PMC3075815

[B49] Pendleton R. G. Parvez F. Sayed M. Hillman R. (2002). Effects of pharmacological agents upon a transgenic model of Parkinson's disease in *Drosophila melanogaster*. J. Pharmacol. Exp. Ther. 300, 91–96. 10.1124/jpet.300.1.91 11752102

[B50] Pesah Y. Pham T. Burgess H. Middlebrooks B. Verstreken P. Zhou Y.. (2004). Drosophila parkin mutants have decreased mass and cell size and increased sensitivity to oxygen radical stress. Development 131, 2183–2194. 10.1242/dev.01095 15073152

[B51] Petrucelli L. O'Farrell C. Lockhart P. J. Baptista M. Kehoe K. Vink L.. (2002). Parkin protects against the toxicity associated with mutant α-Synuclein: proteasome dysfunction selectively affects catecholaminergic neurons. Neuron 36, 1007–1019. 10.1016/S0896-6273(02)01125-X 12495618

[B52] Pickrell A. M. Youle R. J. (2015). The roles of PINK1, parkin, and mitochondrial fidelity in parkinson's disease. Neuron 85, 257–273. 10.1016/j.neuron.2014.12.007 25611507 PMC4764997

[B53] Polymeropoulos M. H. Lavedan C. Leroy E. Ide S. E. Dehejia A. Dutra A.. (1997). Mutation in the α-synuclein gene identified in families with Parkinson's disease. Science. 276, 2045–2047. 10.1126/science.276.5321.2045 9197268

[B54] Poole A. C. Thomas R. E. Andrews L. A. McBride H. M. Whitworth A. J. Pallanck L. J. (2008). The PINK1/Parkin pathway regulates mitochondrial morphology. Proc. Natl. Acad. Sci. U. S. A. 105, 1638–1643. 10.1073/pnas.0709336105 18230723 PMC2234197

[B55] Pramstaller P. P. Schlossmacher M. G. Jacques T. S. Scaravilli F. Eskelson C. Pepivani I.. (2005). Lewy body Parkinson's disease in a large pedigree with 77 Parkin mutation carriers. Ann. Neurol. 58, 411–422. 10.1002/ana.20587 16130111

[B56] Ray Dorsey E. Elbaz A. Nichols E. Abd-Allah F. Abdelalim A. Adsuar J. C.. (2018). Global, regional, and national burden of Parkinson's disease, 1990–2016: a systematic analysis for the Global Burden of Disease Study 2016. Lancet Neurol. 17, 939–953. 10.1016/S1474-4422(18)30295-3 30287051 PMC6191528

[B57] Sarraf S. A. Raman M. Guarani-Pereira V. Sowa M. E. Huttlin E. L. Gygi S. P.. (2013). Landscape of the PARKIN-dependent ubiquitylome in response to mitochondrial depolarization. Nature 496, 372–376. 10.1038/nature12043 23503661 PMC3641819

[B58] Shahmoradian S. H. Lewis A. J. Genoud C. Hench J. Moors T. E. Navarro P. P.. (2019). Lewy pathology in Parkinson's disease consists of crowded organelles and lipid membranes. Nat. Neurosci. 22, 1099–1109. 10.1038/s41593-019-0423-2 31235907

[B59] Strausfeld N. J. Hirth F. (2013). Deep homology of arthropod central complex and vertebrate basal ganglia. Science (80-.). 340, 157–161. 10.1126/science.1231828 23580521

[B60] Strauss R. (2002). The central complex and the genetic dissection of locomotor behaviour. Curr. Opin. Neurobiol. 12, 633–638. 10.1016/S0959-4388(02)00385-9 12490252

[B61] Tare M. Modi R. M. Nainaparampil J. J. Puli O. R. Bedi S. Fernandez-funez P.. (2011). Activation of JNK signaling mediates amyloid-ß- dependent cell. Death. 6, 1–12. 10.1371/journal.pone.0024361 21949710 PMC3173392

[B62] Tito A. J. Cheema S. Jiang M. Zhang S. (2016). A simple one-step dissection protocol for whole-mount preparation of adult drosophila brains. J. Vis. Exp. 2016, 55128. 10.3791/55128-v 27929474 PMC5226341

[B63] Trinh K. Andrews L. Krause J. Hanak T. Lee D. Gelb M.. (2010). Decaffeinated coffee and nicotine-free tobacco provide neuroprotection in Drosophila models of Parkinson's disease through an NRF2-dependent mechanism. J. Neurosci. 30, 5525–5532. 10.1523/JNEUROSCI.4777-09.2010 20410106 PMC3842467

[B64] Trinh K. Moore K. Wes P. D. Muchowski P. J. Dey J. Andrews L.. (2008). Induction of the phase II detoxification pathway suppresses neuron loss in Drosophila models of Parkinson's disease. J. Neurosci. 28, 465–472. 10.1523/JNEUROSCI.4778-07.2008 18184789 PMC6670551

[B65] Van Rompuy A. S. Oliveras-Salvá M. Van Der Perren A. Corti O. Van Den Haute C. Baekelandt V. (2015). Nigral overexpression of alpha-synuclein in the absence of parkin enhances alpha-synuclein phosphorylation but does not modulate dopaminergic neurodegeneration. Mol. Neurodegener. 10, 1–14. 10.1186/s13024-015-0017-8 26099628 PMC4477319

[B66] Wang C. Lu R. Ouyang X. Ho M. W. L. Chia W. Yu F.. (2007). Drosophila overexpressing parkin R275W mutant exhibits dopaminergic neuron degeneration and mitochondrial abnormalities. J. Neurosci. 27, 8563–8570. 10.1523/JNEUROSCI.0218-07.2007 17687034 PMC6672933

[B67] Wang X. Becker K. Levine N. Zhang M. Lieberman A. P. Moore D. J.. (2019). Pathogenic alpha-synuclein aggregates preferentially bind to mitochondria and affect cellular respiration. Acta Neuropathol. Commun. 7, 41. 10.1186/s40478-019-0696-4 30871620 PMC6419482

[B68] White K. E. Humphrey D. M. Hirth F. (2010). The dopaminergic system in the aging brain of Drosophila. Front. Neurosci. 4, 1–12. 10.3389/fnins.2010.00205 21165178 PMC3002484

[B69] Whitworth A. J. Theodore D. A. Greene J. C. Bene,š H. Wes P. D. Pallanck L. J. (2005). Increased glutathione S-transferase activity rescues dopaminergic neuron loss in a Drosophila model of Parkinson's disease. Proc. Natl. Acad. Sci. U. S. A. 102, 8024–8029. 10.1073/pnas.0501078102 15911761 PMC1142368

[B70] Wilkaniec A. Lenkiewicz A. M. Babiec L. Murawska E. Jeśko H. M. Cieślik M.. (2021). Exogenous alpha-synuclein evoked parkin downregulation promotes mitochondrial dysfunction in neuronal cells. implications for Parkinson's disease pathology. Front. Aging Neurosci. 13, 1–21. 10.3389/fnagi.2021.591475 33716707 PMC7943853

[B71] Wilkaniec A. Lenkiewicz A. M. Czapski G. A. Jeśko H. M. Hilgier W. Brodzik R.. (2019). Extracellular alpha-synuclein oligomers induce parkin s-nitrosylation: relevance to sporadic Parkinson's disease etiopathology. Mol. Neurobiol. 56, 125–140. 10.1007/s12035-018-1082-0 29681024 PMC6334739

[B72] Wood-Kaczmar A. Gandhi S. Wood N. W. (2006). Understanding the molecular causes of Parkinson's disease. Trends Mol. Med. 12, 521–528. 10.1016/j.molmed.2006.09.007 17027339

[B73] Yan C. Liu J. Gao J. Sun Y. Zhang L. Song H.. (2019). IRE1 promotes neurodegeneration through autophagy-dependent neuron death in the Drosophila model of Parkinson's disease. Cell Death Dis. 10. 10.1038/s41419-019-2039-6 31641108 PMC6805898

[B74] Yang Y. Nishimura I. Imai Y. Takahashi R. Lu B. (2003). Parkin suppresses dopaminergic neuron-selective neurotoxicity induced by Pael-R in Drosophila. Neuron 37, 911–924. 10.1016/S0896-6273(03)00143-0 12670421

[B75] Yokochi M. (1997). Familial juvenile parkinsonism. Eur. Neurol. 38, 29–33. 10.1159/000113440 9276198

[B76] Yu W. Sun Y. Guo S. Lu B. (2011). The PINK1/Parkin pathway regulates mitochondrial dynamics and function in mammalian hippocampal and dopaminergic neurons. Hum. Mol. Genet. 20, 3227–3240. 10.1093/hmg/ddr235 21613270 PMC3140825

[B77] Zhang L. Karsten P. Hamm S. Pogson J. H. Mü ller-Rischart A. K. Exner N.. (2013). TRAP1 rescues PINK1 loss-of-function phenotypes. Hum. Mol. Genet. 22, 2829–2841. 10.1093/hmg/ddt132 23525905 PMC3690968

