# Supplementary material for: Analysis of α-syn and parkin interaction in mediating neuronal death in Drosophila model of Parkinson's disease
Source: Front Cell Neurosci. 2024 Jan 4;17:1295805. doi: 10.3389/fncel.2023.1295805 (PMC10794313; doi:10.3389/fncel.2023.1295805)
Supplement: Supplementary file 1 [file Data_Sheet_1.PDF]

Supplementary Figure Legends

Suppl. figure 1. Immunohistochemistry showing α-synuclein expression in DA neurons of (A) *SNCA* transgene, (B) Double transgene: *parkin* downregulation with *SNCA* (*Parkin^{IR}; SNCA*) and (C) *wild type-parkin* with *SNCA* (*UAS-SNCA; UAS-parkin*).

Suppl. figure 2. *WT-Parkin* overexpression with *SNCA* overexpression in DA neurons restores the locomotor functions: Climbing assay indicates loss of climbing ability with age in flies. A total of 30 (N=30) flies were used per genotype and 0 flies (n=10) were used for the climbing assay. Data is represented as mean with SEM. Statistical analysis were performed using Two-way ANOVA followed by Tukey's multiple comparison test. P value: *(.033), ** (.002), *** (<0.001), not significant (.12).

Suppl. figure 3. *SNCA*-induced mitochondrial morphology defects is independent of parkin: Western blot of mitochondria fractionation probed for parkin, ATP synthase and Beta-Tubulin (A) and (A') quantification showing reduction in parkin level in mitochondrial and increase in cytosolic fraction. 50 flies (n=50) were used for fractionation. Data is represented as mean with SEM. Statistical analysis were performed using Two-way ANOVA followed by Tukey's multiple comparison test. P value: *(.033), ** (.002), *** (<0.001), not significant (.12).

Suppl. figure 4. Representative cropped images of PPL1 cluster DA neurons showing altered mitochondria morphology in *SNCA*, *Parkin^{IR} and Parkin^{IR}; SNCA* in 7day and 21 Days old adult fly brains, from figure 4: (A-D) 7-day and (E-H) 21-day old adult fly brains showing mito-GFP. Control brains showing mitoGFP at (A) 7-day and (E) 21-day. *SNCA* overexpressing flies show mitoGFP (B) in 7-day and in (F) 21-day. *Parkin^{IR}* expressing flies show mitoGFP in (C) 7-day and in (G) 21-day. *Parkin^{IR}; SNCA* expressing flies show mitoGFP in (D) 7-day and (H) 21-day.
