# Supplementary figures and images for: Analysis of α-syn and parkin interaction in mediating neuronal death in Drosophila model of Parkinson's disease
Source: Front Cell Neurosci. 2024 Jan 4;17:1295805. doi: 10.3389/fncel.2023.1295805 (PMC10794313; doi:10.3389/fncel.2023.1295805)

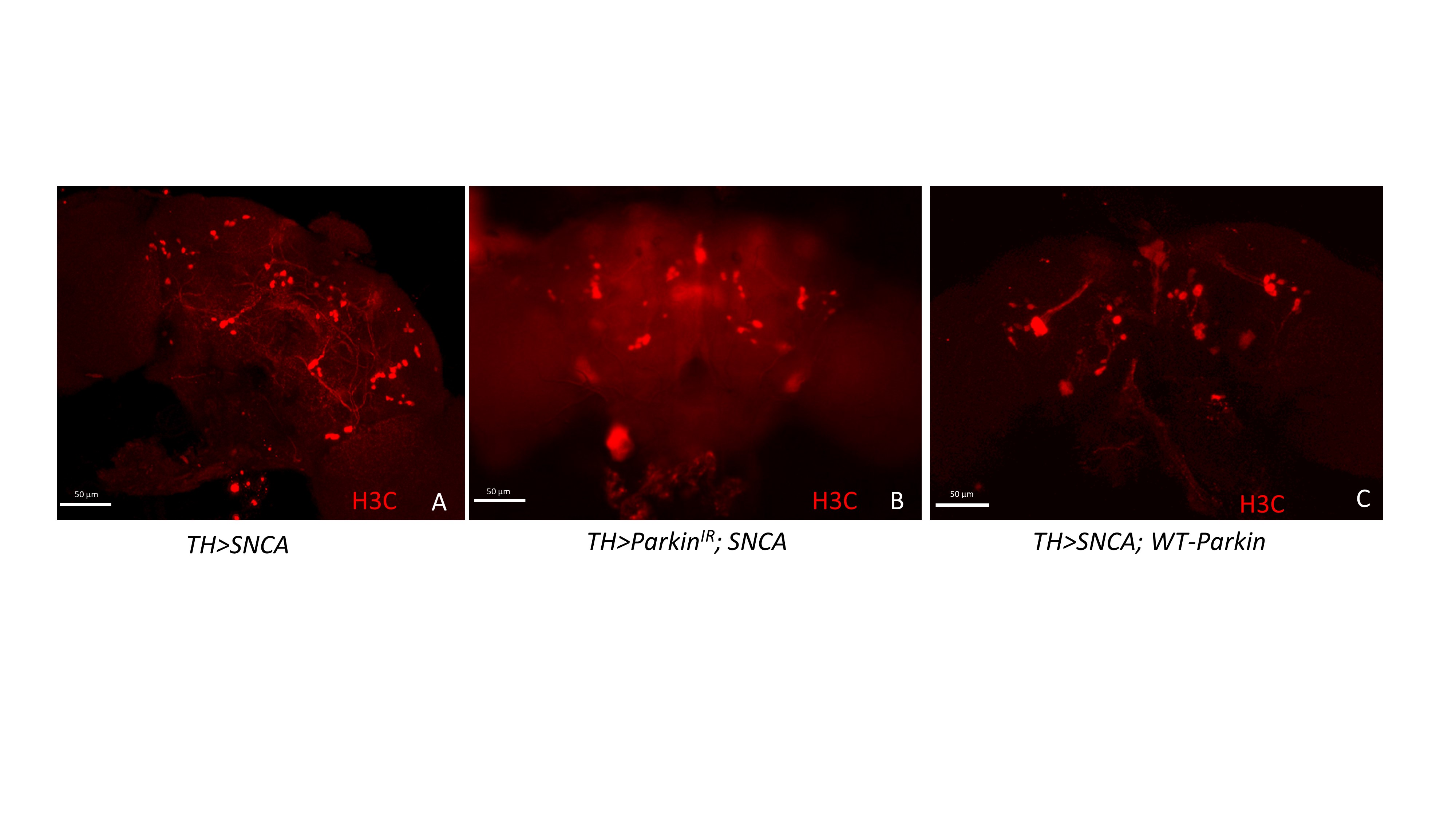

Supplement: Supplementary file 2 [file Image_1.JPEG]

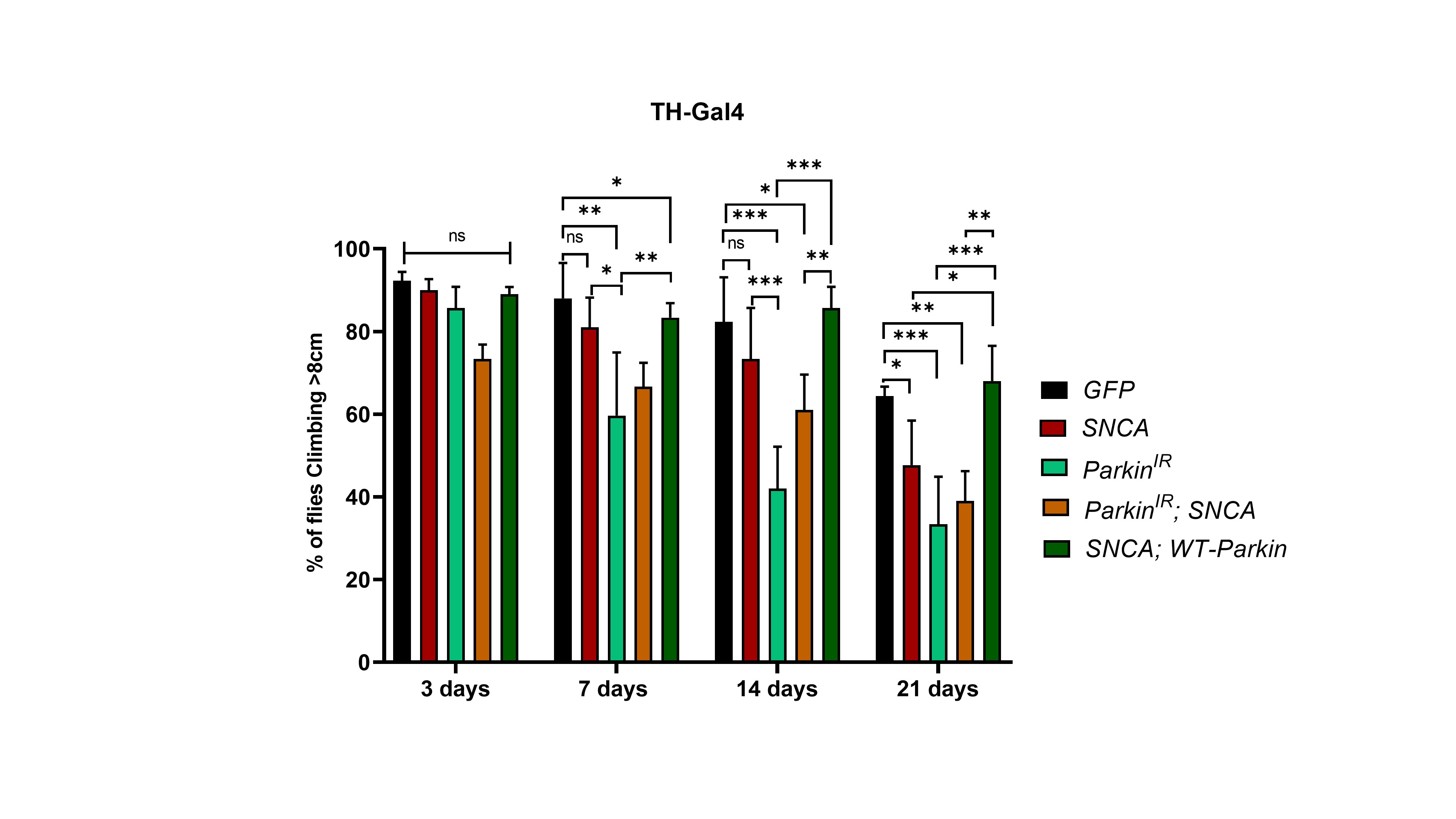

Supplement: Supplementary file 3 [file Image_2.JPEG]

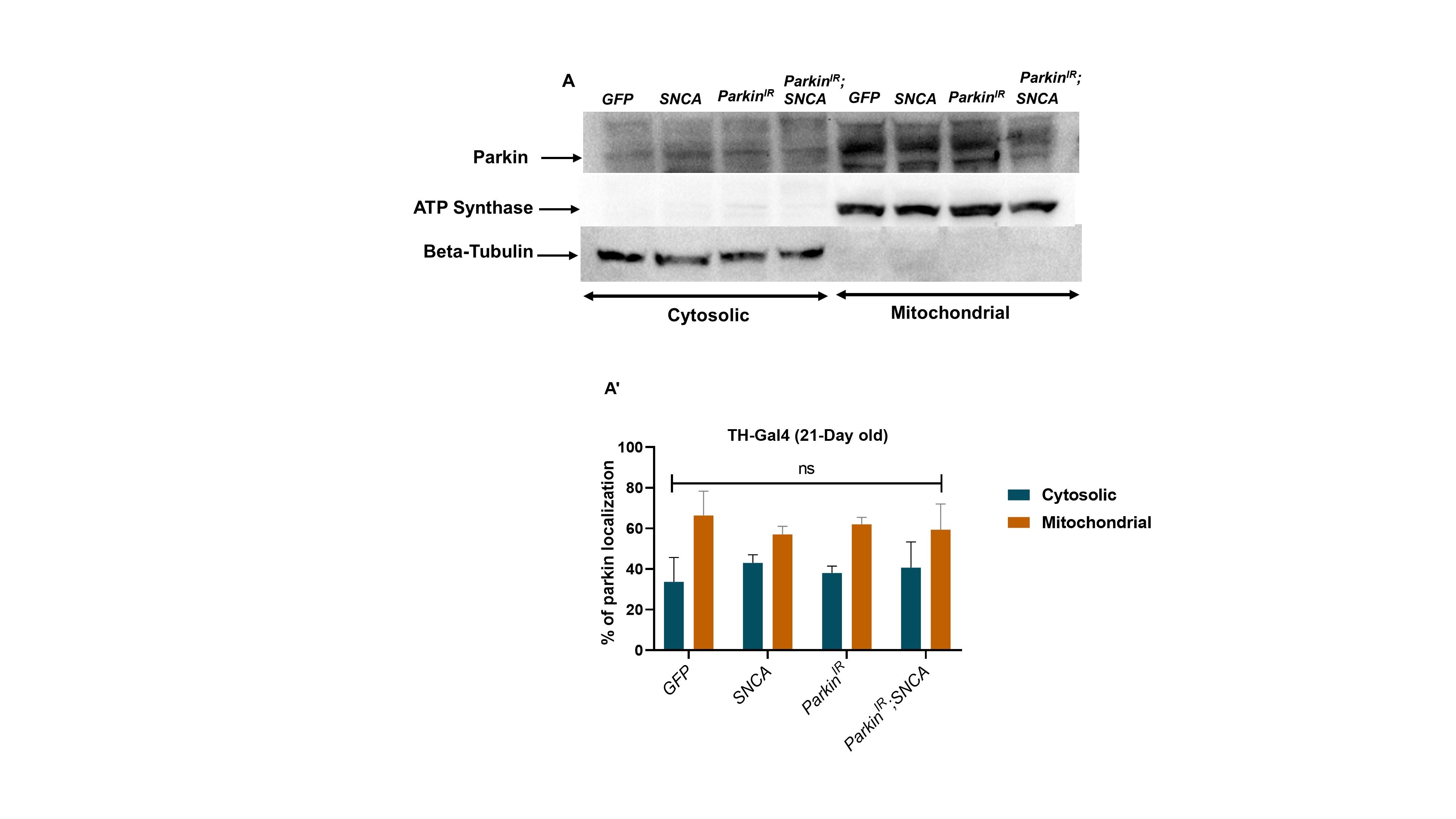

Supplement: Supplementary file 4 [file Image_3.JPEG]

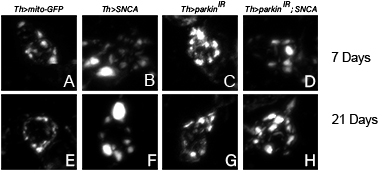

Supplement: Supplementary file 5 [file Image_4.JPEG]
